# Degree Centrality of a Brain Network Is Altered by Stereotype Threat: Evidences From a Resting-State Functional Magnetic Resonance Imaging Study

**DOI:** 10.3389/fpsyg.2021.705363

**Published:** 2021-08-31

**Authors:** Xin Wu, Yufang Zhao

**Affiliations:** ^1^Faculty of Psychology, Southwest University, Chongqing, China; ^2^School of Psychology, Xinxiang Medical University, Xinxiang, China

**Keywords:** stereotype threat, rest-state fMRI, degree centrality, functional network, stereotype

## Abstract

Previous studies have found the effects of stereotype threat (ST) on cognitive processes, emotions, and motivations which could account for the underperformance in domain tasks. Efficient brain function does not require the function of different brain regions during specific tasks, but it does require the brain networks on which information is transported. Based on these, the effects of ST on the degree centrality under the resting state of brain regions related to these processes were investigated under math-related ST. The results showed that RSDC was decreased in the left hippocampus and left middle occipital gyrus (MOC), while RSDC was increased in the left precuneus, the right angular gyrus (AG), and the right superior parietal gyrus (SPG) under ST. Interestingly, we also found that the right-left anterior temporal lobe (ATL) and the right hippocampus were negatively correlated with manipulation check (MC) score in the ST group, while the right-left ATL and the right hippocampus were positively correlated with MC score in the control group. These results might reflect those individuals who attempted to inhibit the negative emotions induced by the negative stereotypes under ST conditions while increasing the self-relevant processes by retrieving episodic memory or autobiographical memory.

## Introduction

Stereotype threat (ST) refers to the feeling of threat which is induced when individuals are worried that their behavior might confirm a stereotype or stigmatize the social identity of their in-group (Schmader and Johns, 2003; Jamieson and Harkins, 2007; Wout et al., 2009). Based on this, it has been confirmed that the negative stereotypes about a social identity could add pressure or concern and lead to underperformance in domain tasks (Steele and Aronson, 1995; Steele, 1997). Although numerous studies show mechanisms of underperformance in domain tasks under ST (e.g., Martens et al., 2006; Wraga et al., 2007; Johns et al., 2008; Krendl et al., 2008), there are only two studies that attempted to uncover the neural mechanisms of ST by using functional magnetic resonance imaging (fMRI) (Wraga et al., 2007; Krendl et al., 2008). Thus, it is helpful to learn more about the neural basis of ST by using the resting-state fMRI (RS-fMRI) to investigate the effects of ST on the degree centrality in this study.

It has been suggested that the negative stereotype could be threatened when it is self-relevant (Steele, 1997). According to the integrated process model of ST, ST first induces an imbalance among the concepts of in-group, the ability of domain tasks, and self, which also implies that ST must be self-relevant (Schmader et al., 2008; Liu et al., 2021). Thus, when the ability of domain tasks is threatened by ST information, it can lead to the processes of self-doubt and self-validating the stereotype (Steele and Aronson, 1995), where negative thinking (Cadinu et al., 2005) and negative emotions (Johns et al., 2008) are further generated. To reduce the detrimental effects of negative thinking and negative emotions on performance, individuals attempt to suppress them, which reduces the working memory capacity required by the performance of domain tasks (Schmader and Johns, 2003; Johns et al., 2008).

Considering the effects of ST on self-relevant processes, negative emotions, and working memory capacity, the brain function related to them might be influenced by ST. For example, it has been found that the brain regions related to social emotion [e.g., angular gyrus (AG), left parietal cortex, and prefrontal cortex] were more active when a domain task was performed under an ST condition (Krendl et al., 2008). Wraga et al. (2007) also found that the brain regions associated with emotional load (e.g., medial prefrontal cortex, anterior cingulate cortex, and amygdala) were activated when a domain task was performed under an ST condition. Moreover, Forbes and Leitner (2014) found that negative feedback to the performance of domain tasks elicited a larger P100 component when individuals were under an ST condition, confirming that the working memory capacity is easily taxed by ST information. Mangels et al. (2012) found that the negative feedback related to event-related potential components is influenced by ST, implying that individuals are prone to disengage from domain tasks influenced by negative emotions.

Efficient brain function does not require the functioning of different brain regions during specific tasks, but it does require information being transported between them (Van Den Heuvel and Hulshoff Pol, 2010). Although some brain regions have been confirmed to be associated with ST, the brain network being influenced by ST is not clear. RS-fMRI can use the blood-oxygen-level-dependent (BOLD) signal of spontaneous activations during low-frequency fluctuations (0.01–0.1 Hz) to depict brain networks (Lowe et al., 2000; Van Den Heuvel and Hulshoff Pol, 2010). RS-fMRI degree centrality (RSDC) is a graph theory-based network analysis of the number of edges connecting to a node or the node strength for a given node (a voxel) in a whole-brain network (Zuo et al., 2012; Li et al., 2016). It has been found that RSDC has test-retest reliability and high sensitivity (Zuo and Xing, 2014) and that it can find the hubs of brain networks and provide functional connectivity of the entire brain (Zhang et al., 2019).

In previous studies (e.g., Schmader and Johns, 2003; Johns et al., 2008; Schmader et al., 2008), the female math ST was widely used to study the ST effect and had common mechanisms with other ST to some extent. Thus, the current study aimed to investigate the effects of ST on RSDC under math ST. According to the results of ST studies (Martens et al., 2006; Wraga et al., 2007; Johns et al., 2008; Krendl et al., 2008), we speculated that the RSDC of brain regions related to the regulation of social emotions (e.g., the medial prefrontal cortex and anterior cingulate cortex) might be increased. Considering the roles of the hippocampus in the generation of stressful responses (Buss et al., 2007; McEwen and Gianaros, 2010; Tottenham and Sheridan, 2010), we hypothesized that the RSDC of the hippocampus might be decreased under ST, which might make individuals more prone to experience stress-based arousal. Except in this region, due to the self-relevant processes being increased by ST (Steele and Aronson, 1995), we thought that the RSDC of the brain regions related to self-memory should be increased. Therefore, we further speculated that the RSDC of the brain regions, such as the right posterior parietal regions (PPC), related to the retrieval of episodic memory or autobiographical memory might be increased (Cabeza, 2008; Cabeza et al., 2011).

## Methods

### Subjects

Forty-eight female undergraduates (25 in the ST group and 23 in the control group), aged 18–26 years (mean age 20.75 ± 1.79 years), participated in the experiment (as shown in Table 1). All the subjects gave written informed consent, were right-handed, had no current or past neurological or psychiatric illness, and had a normal or corrected-to-normal vision.

**Table 1:** The demographic information of Stereotype threat (ST) and control subjects.

	**ST group**	**Control group**	***T*-value**
Mean age	20.40 ± 1.71	21.13 ± 1.84	−1.43
Sex	25 females	23 females	
Education	14.44 ± 1.26	14.87 ± 1.29	−1.17

### Experimental Materials

#### ST and Control Materials

In the present study, math ST was induced by reading math ST material. This material was introduced to subjects with statements such as “women are bad at math across all cultures.” To make the ST material more compelling, a table was provided which showed the percentage differences between men and women for a math ability test. In the control condition, the subjects were instructed to read a scientific investigation about two fictitious mountain peaks.

#### Math Problem Examples

Three math problems were used as examples in this study. There were three numbers (30, 26, 7) in each math problem. The subjects were asked to calculate whether the result of subtraction of the first and second numbers could be divided by the third number; for example, whether the result of “(30 – 26) ÷ 7 = ?” was an integer.

#### Manipulation Check

Previous studies suggested that self-threat in ST might result from concerns over the evaluation about themselves based on the negative stereotype of their ingroup. Thus, to assess the reliability of the ST manipulation, subjects were asked to rate how strongly they agreed with the statement “I am worried that the experimenters will conclude that women are bad at math based on my performance” on a 7-point scale (from strongly disagree to strongly agree).

#### ST and Control Material Verification

To verify whether the ST materials could induce ST, 112 female subjects (56 subjects each for ST and control groups) were selected from the Southwest University in China. First, the subjects were asked to read ST materials (ST group) or control materials (control group). Then, the subjects were asked to complete 20 math problems similar to the examples. The results showed that the mean accuracies of math problems were 0.589 ± 0.149 for the control group and 0.500 ± 0.137 for the ST group. The mean accuracy of math problem answers was lower for the ST group than for the control group [*t*_(110)_ = 3.286, *p* = 0.001].

### Experimental Procedure

When the participants arrived individually at the magnetic resonance imaging (MRI) lab, a male experimenter greeted them and instructed them, and then they were led to the MRI scanner by a male scanning technician. When placed into the MRI scanner and prepared, the subject was allowed to adapt to the MRI scanner for ~8 min. Subjects were asked to relax, keep their eyes open, and focus on the “+” that appeared at the center of the screen, and not to move their body or head. After the RS-fMRI scanning pre-test, the manipulation materials (ST or control materials) were presented, and the subjects were randomly assigned to the ST or control group based on the materials they read. Thereafter, a male experimenter told the subjects that they had about 8 min to prepare for the math aptitude test and presented the three examples of the math problem. The instructions for the post-test RS-fMRI scanning were the same as those used for the pre-test. Each RS-fMRI included 242 scans with 484 s in duration. When the RS-fMRI scanning was over, the male experimenter informed the subjects that there were 20 math problems to be completed and asked them to complete the MC inside the scanner within 3 min. When the MC was completed, the subjects were informed that the experiment was over.

### MRI Image Acquisition and Pre-Processing

#### MRI Image Acquisition

The whole-brain RS-fMRI images were acquired from a Siemens 3T scanner (MAGENTOM Trio, a Tim system) with a gradient-echo echo-planar imaging sequence: echo time (TE) = 30 ms; repetition time (TR) = 2,000 ms; flip angle = 90°; slices = 32; slice thickness = 3.0 mm; slice gap = 1 mm; field of view (FOV) = 220 × 220 mm; resolution matrix = 64 × 64; in-plane resolution = 3.4 × 3.4 mm; interslice skip = 0.99 mm. For each subject, 242 functional images were acquired.

In addition, high-resolution T1-weighted anatomical images were acquired using a magnetization-prepared, rapid gradient echo sequence (TR = 1,900 ms; TE = 2.52 ms; inversion time = 900 ms; flip angle = 9°; resolution matrix = 256 × 256; slices = 176; thickness = 1.0 mm; voxel size = 1 × 1 × 1 mm).

#### Image Pre-Processing

The RS-fMRI images were pre-processed using statistical parametric mapping software (SPM8, http://www.fil.ion.ucl.ac.uk/spm) and a toolbox for Data Processing and Analysis for Brain Imaging (DPABI: http://rfmri.org/dpabi) in MATLAB 8.1.0 (http://cn.mathworks.com/). First, the DICOM data were converted to NIFTI images, and the first 10 images were discarded. Then, the remaining images were slice timed and realigned. A head motion correction was then performed to estimate and modify head movements. The various covariates, including white matter, cerebrospinal fluid, and the Friston 24-parameter, were regressed out of the data to reduce the potential impact of physiological artifacts. Then the anatomical images were co-registered to the mean functional image and were subsequently segmented. Thereafter, all the functional images were normalized to the Montreal Neurological Institute (MNI) space of 3 × 3 × 3 mm voxel sizes using the segmented data. The normalized images were spatially smoothed with a Gaussian kernel having a full width at half maximum (FWHM) of 8 mm. Finally, linear trends were removed, and the images were temporally band-pass filtered (0.01–0.08 Hz) to reduce low-frequency drift and high-frequency noise.

### Degree Centrality Analysis

#### The Calculation of RSDC

The RSDC was calculated using DPABI. The algorithm for RSDC has been reported previously (Zuo et al., 2012) and can be briefly summarized as follows. First, the time series for each voxel was extracted from the pre-processed RS-fMRI data to calculate a correlation matrix using the temporal Pearson's correlation of the time series between certain voxels and others. Then, fully connected binary and weighted graphs were built with a threshold of correlation *r* = 0.25. In the binary graph, the value was 1 if the correlation between two voxels was larger than the threshold; otherwise, the value was 0. In the weighted graph, the value was the correlation if the correlation between two voxels was larger than the threshold; otherwise, the value was 0. According to the adjacency matrix of the graph, the RSDC was calculated for each voxel by the addition of the correlations of each voxel. Finally, the values in each voxel were transformed to *z*-values using the Fisher *z*-transformation to improve normality.

#### Mixed-Effect Analysis

After the RSDC *z*-values for the pre-test and post-test, RS-fMRIs of the two groups were calculated, we performed a whole-brain 2 (test: pre-test vs. post-test) × 2 (ST: ST group vs. control group) mixed-effect analysis for binary and weighted graphs. The statistical criterion was set at a Gaussian random field (GRF) corrected threshold (*p* < 0.001 at the voxel level, and *p* < 0.05 at the cluster level) for the main effect of the test, the main effect of ST, and the interaction effect between ST and test. According to the results, the peak coordinate of each significant cluster was used to create a spherical region of interest (ROI) with an 8-mm radius, and the RSDC values of each ROI were extracted by using the “ROI Signal Extractor” of DPABI. To analyze the sample effect of the interaction effect, the analysis of covariance was used to compare the mean RSDC of each ROI in the post-test between ST and control groups, where the RSDC values in the pre-test were seen as covariables. Finally, the correlations between each ROI and MC score were analyzed by using Pearson's correlation, where the threshold was *p* < 0.05 with Bonferroni correction applied.

#### Interaction Between ST and MC Score Predicting RSDC

To further analyze the relationships between RSDC and MC score, the post-test RSDC values for ST and control groups were input to the SPM to analyze the interaction between the MC score and ST by setting the MC score as the covariate in the two-sample *t*-test of SPM 12. This method has been successfully employed in previous studies to analyze the interaction between variables and the index of structural and functional MRI (Takeuchi et al., 2013; Li et al., 2014, 2018; Wei et al., 2015). The interactions between ST and MC scores were assessed by using *t*-contrasts [(1, −1) or (−1, 1)], where the statistical inference was *p* < 0.005 at the voxel level with GRF-corrected *p* < 0.05 at the cluster level. Based on the significant results, the clusters that showed interactions were saved as masks, and their RSDC values were extracted by using the “ROI Signal Extractor” of DPABI. Finally, to analyze the modulating roles of ST on the relationships between the post-test RSDC of these regions and the MC score, Model 1 of PROCESS 3.0 (www.guilford.com/p/hayes3) was used.

## Results

### Results of the MC

The results showed that the MC score for the ST group was 4.52 ± 1.58, while the MC score for the control group was 3.47 ± 1.31. The two-sample *t*-test analysis showed that the mean MC score for the ST group was significantly higher than that of the control group [*t*_(46)_ = 2.47, *p* = 0.017].

### Results of RSDC Analysis

#### Mixed-Effect Analysis of the Binary Graph

**Figure 1:**
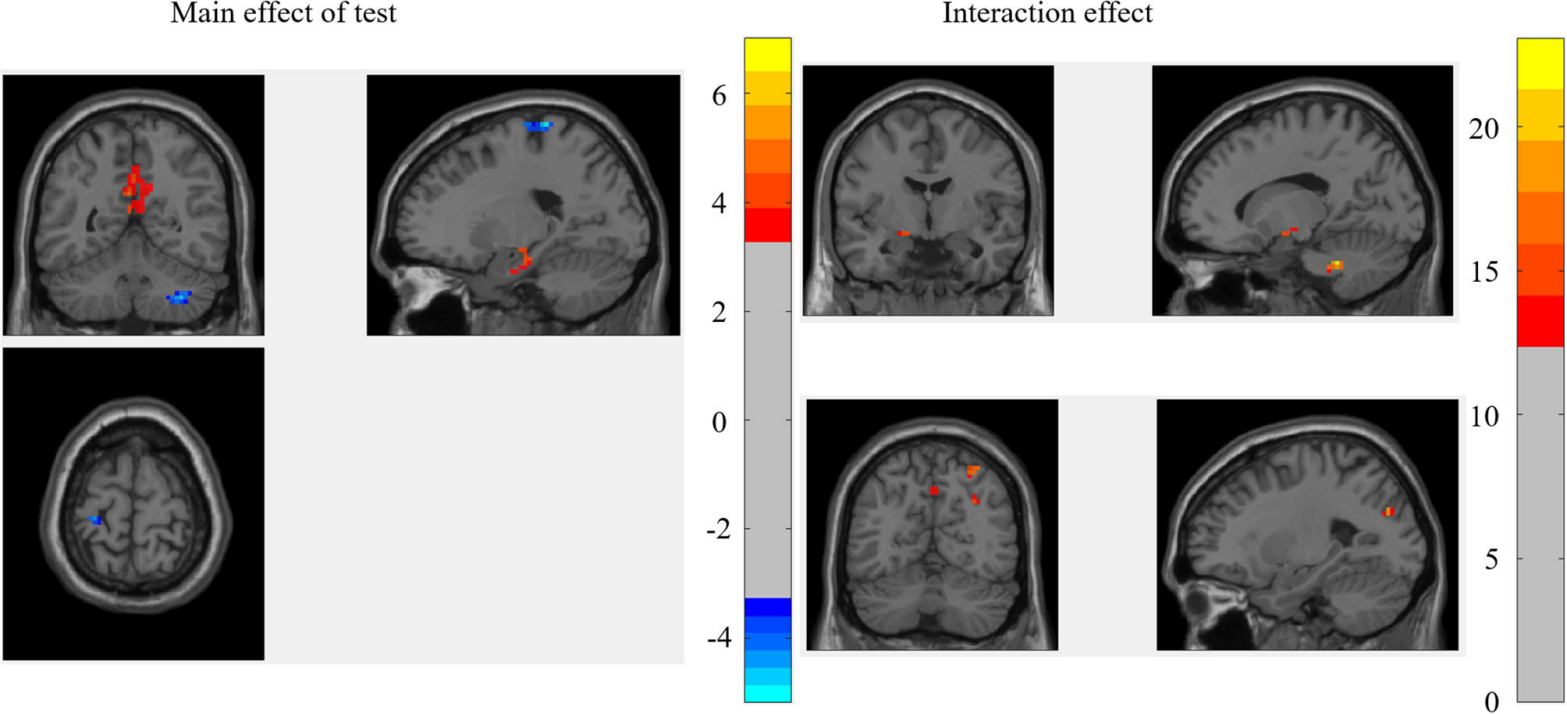
The brain regions that were significant in the mixed-effect analysis of the binary graph.

The results of 2 (test: pre-test vs. post-test) × 2 (ST: ST group vs. control group) mixed-effect analysis for the binary graph showed that the main effect of the test was significant in the left hippocampus, middle cingulate gyrus (MCG), right cerebellum, and left precentral gyrus (PCG), while the interaction between the test and ST was significant in the left cerebellum anterior lobe, left hippocampus, left precuneus, and left MOC (as shown in Figure 1; Table 2).

**Table 2:** The brain regions that were significant in the mixed-effect analysis of the binary graph.

**Cluster size**	**Brain region**	**Cohen's *f*^**2**^**	**F/T**	**Peak**	**Correlated with MC score**	
**(mm^**3**^)**	**Labels**		**Value**	**(x, y, z)**	**ST**	**Control**
**Main effects of test**						
7,047	Left hippocampus	1.073	*T* = 7.03	6, 0, −12	0.202	−0.005
3,996	Middle cingulate gyrus	0.541	*T* = 4.99	0, −45, 36	0.093	−0.026
1,755	Right cerebellum	0.492	*T* = −3.28	39, −54, −42	−0.033	−0.240
1,296	Left precentral gyrus	0.586	*T* = −3.31	−15, −18, 78	−0.002	−0.003
**Main effects of ST**						
No significant voxel was found						
**Interaction**						
513	Cerebellum	0.459	*F* = 21.11	−15, −42, −33	0.096	0.141
378	Right superior parietal gyrus	0.399	*F* = 18.36	24, −66, 57	−0.179	−0.226
324	Left precuneus	0.361	*F* = 16.67	−3, −66, 48	0.153	−0.082
297	Left middle occipital gyrus	0.414	*F* = 19.08	−27, −75, 27	0.018	−0.031
270	Right AG	0.489	*F* = 22.51	33, −60, 36	0.057	0.315
243	Left hippocampus	0.333	*F* = 15.34	−12, −12, −9	−0.021	0.466

For the brain regions that had a significant main effect of the test, the results showed that the mean RSDC *z*-value only in MCG was higher for the ST group relative to the control group [*F*_(1, 45)_ = 4.883, *p* = 0.032]. Of those brain regions that had significant interactions, the mean RSDC *z*-value in the left cerebellum was lower for the ST group relative to the control group [*F*_(1, 45)_ = 8.484, *p* = 0.006]; the mean RSDC *z*-value in the right superior parietal gyrus (SPG) was higher for the ST group relative to the control group [*F*_(1, 45)_ = 8.453, *p* = 0.006]; the mean RSDC *z*-value in the left precuneus was higher for the ST group relative to the control group [*F*_(1, 45)_ = 8.426, *p* = 0.006]; the mean RSDC *z*-value in the left MOG was higher for the ST group relative to the control group [*F*_(1, 45)_ = 7.522, *p* = 0.009]; the mean RSDC *z*-value in the right AG was higher for the ST group relative to the control group [*F*_(1, 45)_ = 8.976, *p* = 0.004]; and the mean RSDC *z*-value in the left hippocampus was lower for the ST group relative to the control group [*F*_(1, 45)_ = 7.851, *p* = 0.007]. However, the RSDC for these regions was not significantly correlated with the MC score (as shown in Table 2).

#### Mixed-Effect Analysis of the Weighted Graph

**Figure 2:**
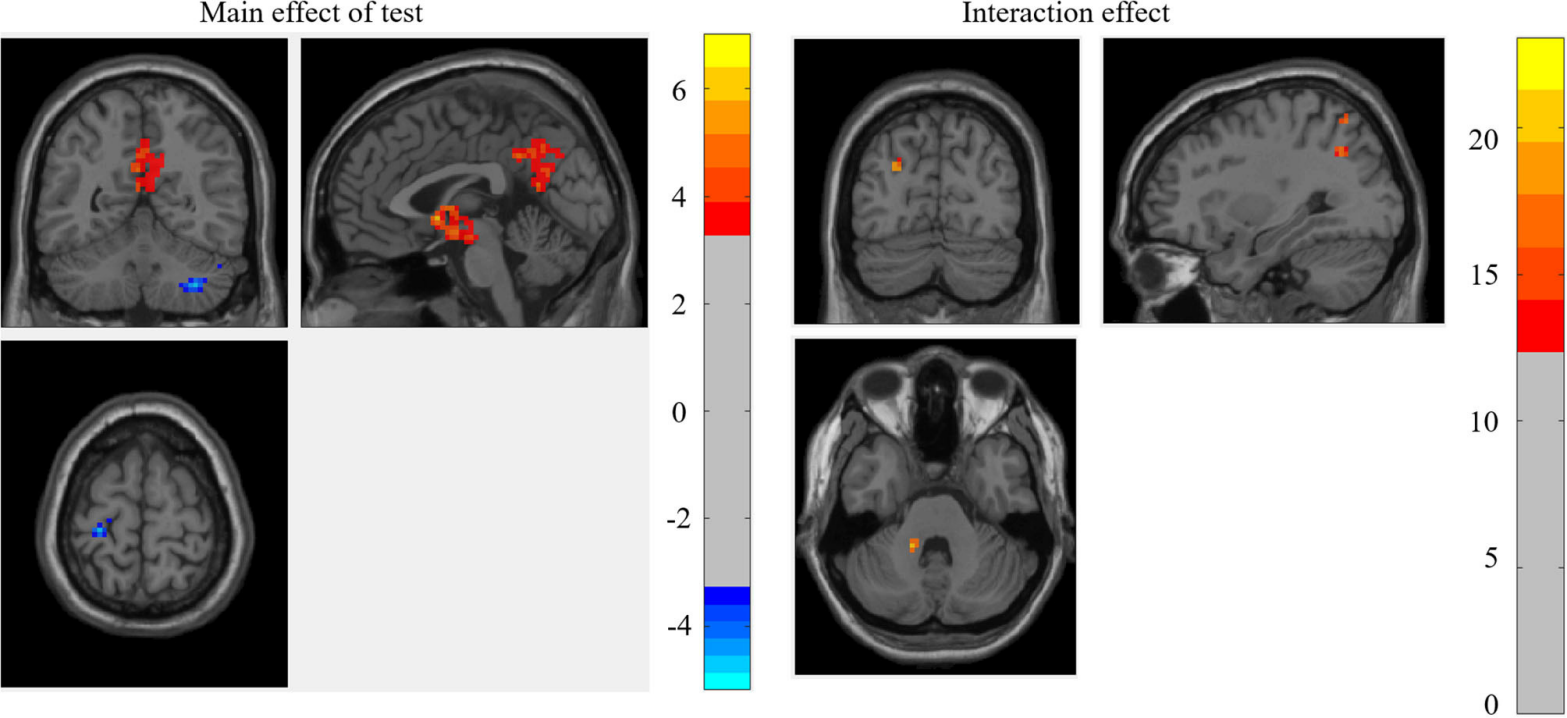
The brain regions that were significant in the mixed-effect analysis of the weighted graph.

The results of whole-brain 2 (test: pre-test vs. post-test) × 2 (ST: ST group vs. control group) mixed-effect analysis for the weighted graph showed that the main effect of the test was significant in the left hippocampus, left MCG, right cerebellum, and left PCG, while the interaction between the test and ST was significant in the left cerebellum anterior lobe, left precuneus, left MCG, right SPG, and right AG (as shown in Figure 2; Table 2).

For the brain regions that had a significant main effect of the test, the results showed that the mean RSDC *z*-value only in MCG was higher for the ST group relative to the control group [*F*_(1, 45)_ = 4.854, *p* = 0.033]. For the brain regions that had significant interactions, the mean RSDC *z*-value in the left cerebellum was lower for the ST group relative to the control group [*F*_(1, 45)_ = 4.228, *p* = 0.046]; the mean RSDC *z*-value in the right SPG was higher for the ST group relative to the control group [*F*_(1, 45)_ = 7.594, *p* = 0.008]; the mean RSDC *z*-value in the left precuneus was higher for the ST group relative to the control group [*F*_(1, 45)_ = 8.900, *p* = 0.005]; the mean RSDC *z*-value in the left MOG was higher for the ST group relative to the control group [*F*_(1, 45)_ = 7.571, *p* = 0.009]; and the mean RSDC *z*-value in AG was higher for the ST group relative to the control group [*F*_(1, 45)_ = 9.199, *p* = 0.004]. However, the RSDC for these regions was not significantly correlated with the MC score (as shown in Table 3).

**Table 3:** The brain regions that were significant in the mixed-effect analysis of the weighted graph.

**Cluster size**	**Brain region**	**Cohen's *f*^**2**^**	**F/T**	**Peak**	**Correlated with MC score**	
**(mm^**3**^)**	**Labels**		**value**	**(x, y, z)**	**ST**	**Control**
**Main effect of test**						
5,265	Left hippocampus	1.031	*T* = 6.89	6, 0, −12	0.195	−0.015
4,698	Left middle cingulate gyrus	0.547	*T* = 5.02	0, −45, 36	0.057	−0.034
1,890	Cerebellum	0.644	*T* = −3.28	48, −54, −33	−0.048	−0.243
1,647	Left precentral gyrus	0.618	*T* = −3.30	−15, −18, 78	0.008	0.009
**Main effect of ST**						
No significant voxel was found						
**Interaction**						
459	Cerebellum	0.447	*F* = 20.57	−15, −42, −33	0.101	0.134
297	Left middle occipatal gyrus	0.421	*F* = 19.39	−27, −75, 27	0.012	−0.027
297	Left precuneus	0.371	*F* = 17.06	−3, −66, 48	0.128	−0.039
297	Right superior parietal gyrus	0.359	*F* = 16.54	24, −66, 54	−0.132	−0.221
243	Right AG	0.456	*F* = 22.51	33, −60, 36	0.057	0.290

#### The Modulating Roles of ST in the Relationships Between RSDC and MC Score

**Figure 3:**
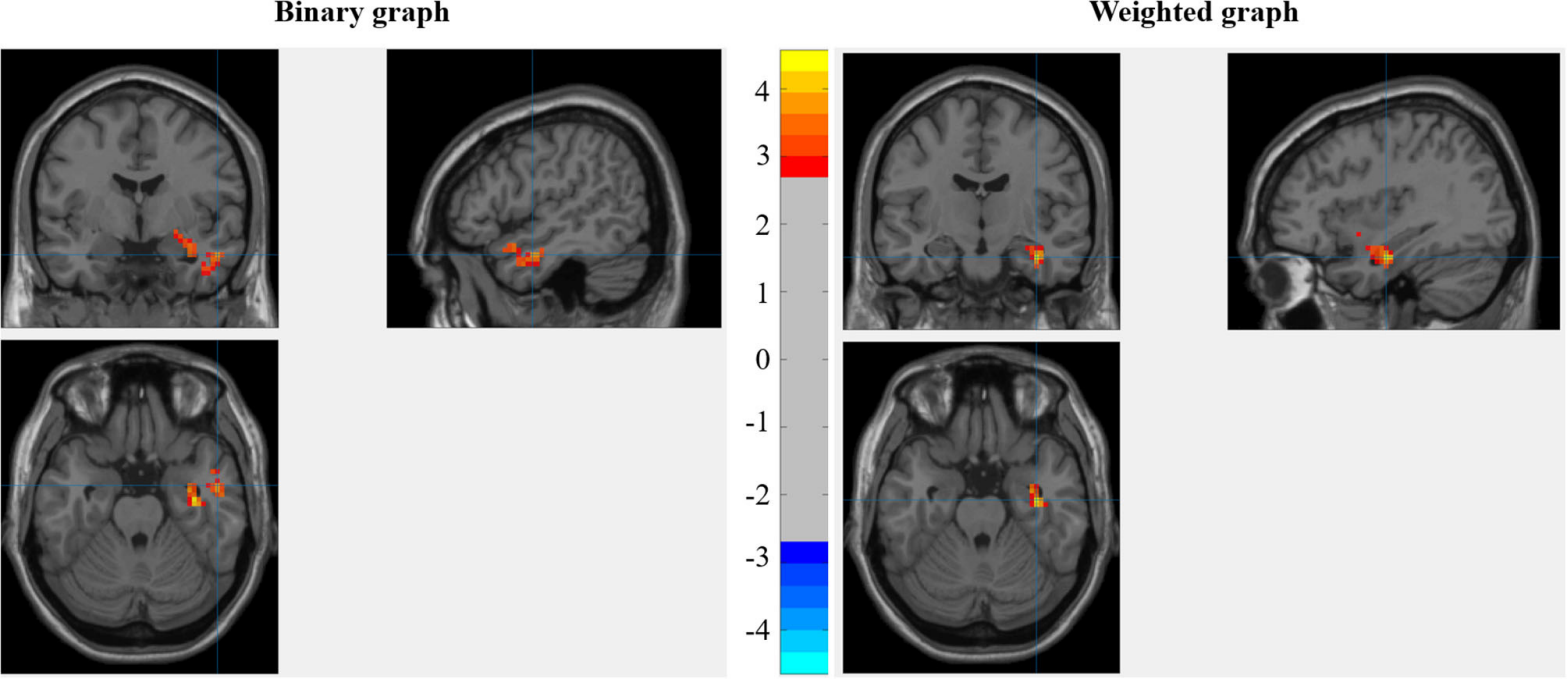
The interaction between stereotype threat (ST) and manipulation check (MC) score when predicting the degree centrality under the resting state (RSDC).

The interaction between ST and MC score was significant in the right anterior temporal lobe (ATL) [peak in (51, −3, −24)] and the right hippocampus/amygdala [peak in (36, −12, −24)] for the binary graph, while only the right hippocampus/amygdala [peak in (36, −12, −24)] showed significant results for the weighted graph (as shown in Figure 3).

**Figure 4:**
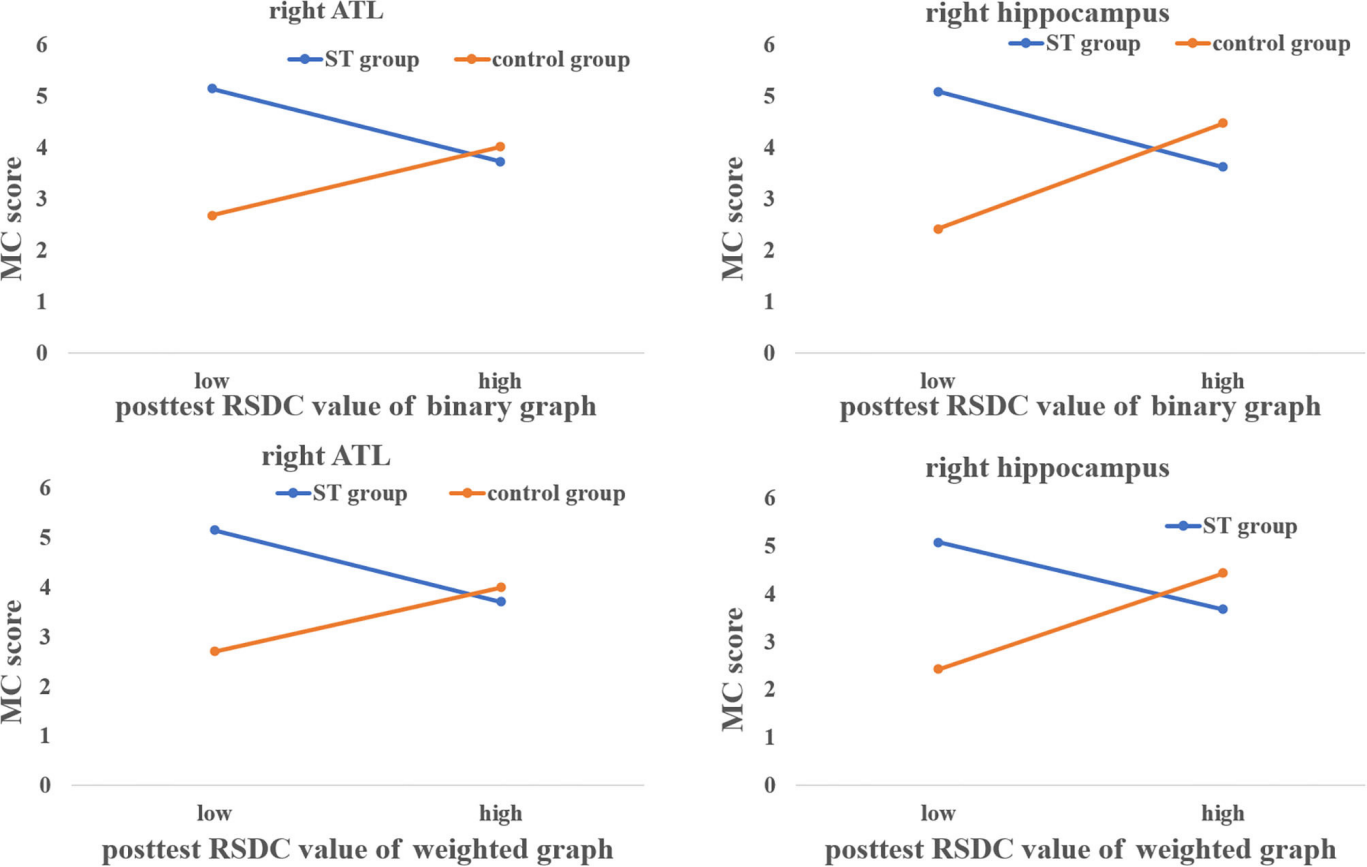
The modulating roles of ST on the relationships between RSDC and MC score.

For the binary graph, the results showed that the interaction between ST and post-test RSDC of the right ATL was significant [Δ*R*^2^ = 0.246, *F*_(1,43)_ = 17.743, *p* < 0.001]. The sample slope test showed that the post-test RSDC for the ST group was negatively correlated with the MC score (β = −0.548, *t* = −2.438, *p* = 0.019), while the post-test RSDC for the ST group was positively correlated with the MC score (β = 0.517, *t* = 3.234, *p* = 0.002). The interaction between ST and post-test RSDC of the right hippocampus was significant [Δ*R*^2^ = 0.300, *F*_(1,43)_ = 23.11, *p* < 0.001]. The sample slope test showed that the post-test RSDC of the right hippocampus was negatively correlated with the MC score in the ST group (β = −0.416, *t* = −2.470, *p* = 0.018), while the post-test RSDC of the hippocampus was positively correlated with the MC score in the control group (β = 0.731, *t* = 3.907, *p* < 0.001) see Figure 4.

For the weighted graph, the results showed that the interaction between ST and post-test RSDC of the right ATL was significant for the weighted graph [Δ*R*^2^ = 0.239, *F*_(1,43)_ = 16.998, *p* < 0.001]. The sample slope test showed that the post-test RSDC for the ST group was negatively correlated with the MC score (β = −0.561, *t* = −2.446, *p* = 0.019), while the post-test RSDC for the ST group was positively correlated with the MC score (β = 0.501, *t* = 3.175, *p* = 0.003). The interaction between ST and post-test RSDC of the right hippocampus was significant [Δ*R*^2^ = 0.295, *F*_(1,43)_ = 22.50, *p* < 0.001]. The sample slope test showed that the post-test RSDC of the right hippocampus was negatively correlated with the MC score in the ST group (β = −0.458, *t* = −2.431, *p* = 0.019), while the post-test RSDC of the hippocampus was positively correlated with the MC score in the control group (β = 0.660, *t* = 3.535, *p* < 0.001) see Figure 4.

## Discussion

In the present study, the effects of ST on the RSDC of brain networks were investigated using RS-fMRI under math ST conditions. The results showed that the RSDC decreased in the left MOG and the left hippocampus, while the RSDC increased in the right MCG, right SPG, right AG, and left precuneus. Furthermore, the results also showed that the right ATL and right hippocampus were negatively correlated with the MC score in the ST group, while the right ATL and the right hippocampus were positively correlated with the MC score in the control group.

It has been found that brain regions in the posteromedial cortex, such as the precuneus and posterior cingulate cortex (PCC), are the hubs of the structural and functional networks of the brain (Hagmann et al., 2008), which means that these regions are highly connected to cortical and subcortical networks (Hagmann et al., 2008; Leech et al., 2012). These characteristics are involved in broad mental processing, such as episodic memory (autobiographical memory), mentalizing (Muscatell et al., 2012), self-referential processing, or self-reflection (Northoff and Bermpohl, 2004; van der Meer et al., 2010), and they allow the brain to acquire information from functionally distinct brain networks easily (Leech et al., 2012). Moreover, the left precuneus is a part of the default mode network (DMN) and plays an important role in self-generated thoughts (Andrews-Hanna et al., 2014; Axelrod et al., 2015). Therefore, we speculated that the increased RSDC in the left precuneus and MCG might reflect that some self-relevant thoughts are generated by retrieving autobiographical memory or episodic memory.

The self-relevant processes under ST could also be reflected by the increased RSDC in the PPC. Previous studies suggested that the PPC can be divided into the dorsal parietal cortex (DPC) and ventral parietal cortex (VPC), where the DPC includes the intraparietal sulcus and SPG, and the VPC includes the supramarginal and angular gyri (Cabeza, 2008; Cabeza et al., 2011). Although both VPC and DPC are involved in the retrieval of autobiographical memory or episodic memory, the DPC is mainly involved in the top-down attention of retrieving these memories, and the VPC is mainly related to the bottom-up attention of these memories that are activated automatically (Cabeza, 2008; Cabeza et al., 2011; Wu et al., 2015). Moreover, the PPC is also located in the DMN and plays an important role in self-generated thoughts (Andrews-Hanna et al., 2014; Axelrod et al., 2015). Therefore, the increased RSDC in PPC for the ST group might further reflect that the self-relevant processes were induced by ST.

It has been suggested that ST increases stress-based arousal, such as increasing the activation of the sympathetic nervous system (Murphy et al., 2007), blood pressure (Blascovich et al., 2001), and cardiovascular responses (Mendes et al., 2002). Results from both animals and humans confirm that the experience of stress has significant negative effects on the hippocampus (Buss et al., 2007; McEwen and Gianaros, 2010; Tottenham and Sheridan, 2010), where glucocorticoids and mineralocorticoids are released to regulate the hypothalamic-pituitary-adrenal (HPA) axis on which physiological processes depend (Henry et al., 1994; Kudielka et al., 2004). The hippocampus is an important brain region for the regulation of stress hormones (Kim and Diamond, 2002; Glover et al., 2010), such as inhibiting the HPA axis through glucocorticoid-mediated negative feedback to terminate the stress response (Sapolsky, 1992; McEwen and Sapolsky, 1995). According to the results, the RSDC of the left hippocampus was decreased, and the RSDC of the right hippocampus was negatively correlated with the MC score under the ST condition. These results might reflect that the importance of the hippocampus in the brain networks was decreased by the ST condition, which might make individuals prone to exhibit stress-based arousal mentioned earlier.

It has been found that the role of the ATL was related to linking person-specific memories to representations of faces at the perception level for face processing (Olson et al., 2007) and is associated with the processes of comprehending the mind of others based on personal memories (Moriguchi et al., 2006). In addition, the ATL has an important role in social concepts (Olson et al., 2013). For example, previous studies have shown that stereotypes (a social concept) are represented by the ATL, which might play an important role in linking certain types of people to specific personality or behavioral traits (Olson et al., 2013). According to the results, the RSDC of the right ATL was negatively correlated to the MC score in the ST group, while the RSDC of the right ATL was positively correlated to the MC score in the control group. These findings reflect that the relative importance of the brain regions related to social concepts is decreased under ST, especially for more threatened individuals. It is dangerous to increase the self-relevant processes related to ST and at the same time decrease the functions of social concepts. Doing so might make individuals prone to be influenced by their stigmatized social identity and discount other beneficial social identities. Consistent with this perspective, a previous study found that learning other beneficial social identities can decrease the effects of ST (Rydell et al., 2009).

## Conclusion

We detected the effects of ST on brain network degree centrality by directly comparing the RSDC between the ST and control groups. These findings expand the knowledge of the neural basis of ST. Specifically, the results suggest that ST decreases the importance of brain regions related to social concepts and stress regulation in the whole brain networks and, at the same time, increases the importance of brain regions associated with self-relevant processes. We acknowledge several limitations of the study. First, the performance of the math problem was not measured in an RS-fMRI study, and we did not confirm whether the altered RSDCs were related to underperformance under ST. Second, Steele and Aronson (1995) found that ST could lead to some self-concerning processes, such as self-doubts, self-validating the stereotype, and stereotype avoidance. Although we speculate that the altered RSDCs might be related to these mental processes, we could not provide direct evidence for them because behavior related to these processes was not measured. Third, due to this evidence being exclusively from female math ST, future studies should verify whether these results generalize to other STs (e.g., racial ST or ST related to social status). Therefore, we suggest that a sophisticated experiment integrating behavioral, task, and RS-fMRI may be required to reveal the detailed mechanism of ST.

## Data Availability Statement

The datasets generated for this study are available on request to the corresponding author.

## Ethics Statement

The studies involving human participants were reviewed and approved by Brain Imaging Center Institutional Review Board of Southwest China University. The patients/participants provided their written informed consent to participate in this study.

## Author Contributions

XW and YZ designed the experiment and wrote this manuscript. Both authors contributed to the article and approved the submitted version.

## Conflict of Interest

The authors declare that the research was conducted in the absence of any commercial or financial relationships that could be construed as a potential conflict of interest.

## Publisher's Note

All claims expressed in this article are solely those of the authors and do not necessarily represent those of their affiliated organizations, or those of the publisher, the editors and the reviewers. Any product that may be evaluated in this article, or claim that may be made by its manufacturer, is not guaranteed or endorsed by the publisher.

## References

[B1] Andrews-Hanna J. R. Smallwood J. Spreng R. N. (2014). The default network and self-generated thought: component processes, dynamic control, and clinical relevance. Ann. NY Acad. Sci. 1316:29. 10.1111/nyas.12360 24502540 PMC4039623

[B2] Axelrod V. Bar M. Rees G. Yovel G. (2015). Neural correlates of subliminal language processing. Cerebral Cortex 25, 2160–2169. 10.1093/cercor/bhu022 24557638 PMC4494027

[B3] Blascovich J. Spencer S. J. Quinn D. Steele C. (2001). African Americans and high blood pressure: the role of stereotype threat. Psychol. Sci. 12, 225–229. 10.1111/1467-9280.00340 11437305

[B4] Buss C. Lord C. Wadiwalla M. Hellhammer D. H. Lupien S. J. Meaney M. J.. (2007). Maternal care modulates the relationship between prenatal risk and hippocampal volume in women but not in men. J. Neurosci. 27, 2592–2595. 10.1523/JNEUROSCI.3252-06.2007 17344396 PMC6672503

[B5] Cabeza R. (2008). Role of parietal regions in episodic memory retrieval: the dual attentional processes hypothesis. Neuropsychologia 46, 1813–1827. 10.1016/j.neuropsychologia.2008.03.019 18439631 PMC2517132

[B6] Cabeza R. Mazuz Y. S. Stokes J. Kragel J. E. Woldorff M. G. Ciaramelli E.. (2011). Overlapping parietal activity in memory and perception: evidence for the attention to memory model. J. Cogn. Neurosci. 23, 3209–3217. 10.1162/jocn_a_00065 21568633 PMC3518433

[B7] Cadinu M. Maass A. Rosabianca A. Kiesner J. (2005). Why do women underperform under stereotype threat? Evidence for the role of negative thinking. Psychol. Sci. 16, 572–578. 16008792 10.1111/j.0956-7976.2005.01577.x

[B8] Forbes C. E. Leitner J. B. (2014). Stereotype threat engenders neural attentional bias toward negative feedback to undermine performance. Biol. Psychol. 102, 98–107. 10.1016/j.biopsycho.2014.07.007 25063472

[B9] Glover V. O'connor T. G. O'Donnell K. (2010). Prenatal stress and the programming of the HPA axis. Neurosci. Biobehav. Rev. 35, 17–22. 10.1016/j.neubiorev.2009.11.008 19914282

[B10] Hagmann P. Cammoun L. Gigandet X. Meuli R. Honey C. J. Wedeen V. J.. (2008). Mapping the structural core of human cerebral cortex. PLoS Biol. 6, e159. 10.1371/journal.pbio.0060159 18597554 PMC2443193

[B11] Henry C. Kabbaj M. Simon H. Le Moal M. Maccari S. (1994). Prenatal stress increases the hypothalamo-pituitary-adrenal axis response in young and adult rats. J. Neuroendocrinol. 6, 341–345. 10.1111/j.1365-2826.1994.tb00591.x 7920600

[B12] Jamieson J. P. Harkins S. G. (2007). Mere effort and stereotype threat performance effects. J. Pers. Soc. Psychol. 93, 544–564. 10.1037/0022-3514.93.4.544 17892331

[B13] Johns M. Inzlicht M. Schmader T. (2008). Stereotype threat and executive resource depletion: examining the influence of emotion regulation. J. Exp. Psychol. Gen. 137, 691–705. 10.1037/a0013834 18999361 PMC2976617

[B14] Kim J. J. Diamond D. M. (2002). The stressed hippocampus, synaptic plasticity and lost memories. Nat. Rev. Neurosci. 3, 453–462. 10.1038/nrn849 12042880

[B15] Krendl A. C. Richeson J. A. Kelley W. M. Heatherton T. F. (2008). The negative consequences of threat: a functional magnetic resonance imaging investigation of the neural mechanisms underlying women's underperformance in math. Psychol. Sci. 19, 168–175. 10.1111/j.1467-9280.2008.02063.x 18271865

[B16] Kudielka B. M. Schommer N. C. Hellhammer D. H. Kirschbaum C. (2004). Acute HPA axis responses, heart rate, and mood changes to psychosocial stress (TSST) in humans at different times of day. Psychoneuroendocrinology 29, 983–992. 10.1016/j.psyneuen.2003.08.009 15219648

[B17] Leech R. Braga R. Sharp D. J. (2012). Echoes of the brain within the posterior cingulate cortex. J. Neurosci. 32, 215–222. 10.1523/JNEUROSCI.3689-11.2012 22219283 PMC6621313

[B18] Li B. Li X. Pan Y. Qiu J. Zhang D. (2018). The relationship between self-enhancing humor and precuneus volume in young healthy individuals with high and low cognitive empathy. Sci. Rep. 8, 1–6. 10.1038/s41598-018-21890-0 29472593 PMC5823885

[B19] Li H. Li L. Shao Y. Gong H. Zhang W. Zeng X.. (2016). Abnormal intrinsic functional hubs in severe male obstructive sleep apnea: evidence from a voxel-wise degree centrality analysis. PLoS ONE 11:e0164031. 10.1371/journal.pone.0164031 27723821 PMC5056709

[B20] Li H. J. Sun J. Z. Zhang Q. L. Wei D. T. Li W. F. Jackson T.. (2014). Neuroanatomical differences between men and women in help-seeking coping strategy. Sci. Rep. 4, 1–5. 10.1038/srep05700 25027617 PMC4099976

[B21] Liu S. Liu P. Wang M. Zhang B. (2021). Effectiveness of stereotype threat interventions: a meta-analytic review. J. Appl. Psychol. 106, 921–949. 10.1037/apl0000770 32772526

[B22] Lowe M. J. Dzemidzic M. Lurito J. T. Mathews V. P. Phillips M. D. (2000). Correlations in low-frequency bold fluctuations reflect cortico-cortical connections. Neuroimage 12, 582–587. 10.1006/nimg.2000.0654 11034865

[B23] Mangels J. A. Good C. Whiteman R. C. Maniscalco B. Dweck C. S. (2012). Emotion blocks the path to learning under stereotype threat. Soc. Cogn. Affect. Neurosci. 7, 230–241. 10.1093/scan/nsq100 21252312 PMC3277368

[B24] Martens A. Johns M. Greenberg J. Schimel J. (2006). Combating stereotype threat: the effect of self-affirmation on women's intellectual performance. J. Exp. Soc. Psychol. 42, 236–243. 10.1016/j.jesp.2005.04.010

[B25] McEwen B. S. Gianaros P. J. (2010). Central role of the brain in stress and adaptation: links to socioeconomic status, health, and disease. Ann. NY Acad. Sci. 1186, 190–222. 10.1111/j.1749-6632.2009.05331.x 20201874 PMC2864527

[B26] McEwen B. S. Sapolsky R. M. (1995). Stress and cognitive function. Curr. Opin. Neurobiol. 5, 205–216. 10.1016/0959-4388(95)80028-X 7620309

[B27] Mendes W. B. Blascovich J. Lickel B. Hunter S. (2002). Challenge and threat during social interactions with White and Black men. Pers. Soc. Psychol. Bull. 28, 939–952. 10.1177/01467202028007007

[B28] Moriguchi Y. Ohnishi T. Lane R. D. Maeda M. Mori T. Nemoto K.. (2006). Impaired self-awareness and theory of mind: an fMRI study of mentalizing in alexithymia. Neuroimage 32, 1472–1482. 10.1016/j.neuroimage.2006.04.186 16798016

[B29] Murphy M. C. Steele C. M. Gross J. J. (2007). Signaling threat: how situational cues affect women in math, science, and engineering settings. Psychol. Sci. 18, 879–885. 10.1111/j.1467-9280.2007.01995.x 17894605

[B30] Muscatell K. A. Morelli S. A. Falk E. B. Way B. M. Pfeifer J. H. Galinsky A. D.. (2012). Social status modulates neural activity in the mentalizing network. Neuroimage 60, 1771–1777. 10.1016/j.neuroimage.2012.01.080 22289808 PMC3909703

[B31] Northoff G. Bermpohl F. (2004). Cortical midline structures and the self. Trends Cogn. Sci. 8, 102–107. 10.1016/j.tics.2004.01.004 15301749

[B32] Olson I. R. McCoy D. Klobusicky E. Ross L. A. (2013). Social cognition and the anterior temporal lobes: a review and theoretical framework. Soc. Cogn. Affect. Neurosci. 8, 123–133. 10.1093/scan/nss119 23051902 PMC3575728

[B33] Olson I. R. Plotzker A. Ezzyat Y. (2007). The enigmatic temporal pole: a review of findings on social and emotional processing. Brain 130, 1718–1731. 10.1093/brain/awm052 17392317

[B34] Rydell R. J. McConnell A. R. Beilock S. L. (2009). Multiple social identities and stereotype threat: imbalance, accessibility, and working memory. J. Pers. Soc. Psychol. 96, 949–966. 10.1037/a0014846 19379029

[B35] Sapolsky R. M. (1992). Stress, the Aging Brain, and the Mechanisms of Neuron Death. Cambridge, MA: The MIT Press.

[B36] Schmader T. Johns M. (2003). Converging evidence that stereotype threat reduces working memory capacity. J. Pers. Soc. Psychol. 85, 440–452. 10.1037/0022-3514.85.3.440 14498781

[B37] Schmader T. Johns M. Forbes C. (2008). An integrated process model of stereotype threat effects on performance. Psychol. Rev. 115, 336–356. 10.1037/0033-295X.115.2.336 18426293 PMC2570773

[B38] Steele C. (1997). A threat in the air: how stereotypes shape intellectual identity and performance. Am. Psychol. 52, 613–629. 10.1037/0003-066X.52.6.613 9174398

[B39] Steele C. M. Aronson J. (1995). Stereotype threat and the intellectual test performance of African Americans. J. Pers. Soc. Psychol. 69, 797–811. 10.1037/0022-3514.69.5.797 7473032

[B40] Takeuchi H. Taki Y. Thyreau B. Sassa Y. Hashizume H. Sekiguchi A.. (2013). White matter structures associated with empathizing and systemizing in young adults. Neuroimage 77, 222–236. 10.1016/j.neuroimage.2013.04.004 23578577

[B41] Tottenham N. Sheridan M. A. (2010). A review of adversity, the amygdala and the hippocampus: a consideration of developmental timing. Front. Hum. Neurosci. 3:68. 10.3389/neuro.09.068.2009 20161700 PMC2813726

[B42] Van Den Heuvel M. P. Hulshoff Pol H. E. (2010). Exploring the brain network: a review on resting-state fmri functional connectivity. Eur. Neuropsychopharmacol. J. Eur. Coll. Neuropsychopharmacol. 20, 519–534. 10.1016/j.euroneuro.2010.03.008 20471808

[B43] van der Meer L. Costafreda S. Aleman A. David A. S. (2010). Self-reflection and the brain: a theoretical review and meta-analysis of neuroimaging studies with implications for schizophrenia. Neurosci. Biobehav. Rev. 34, 935–946. 10.1016/j.neubiorev.2009.12.004 20015455

[B44] Wei D. Du X. Li W. Chen Q. Li H. Hao X.. (2015). Regional gray matter volume and anxiety-related traits interact to predict somatic complaints in a non-clinical sample. Soc. Cogn. Affect. Neurosci. 10, 122–128. 10.1093/scan/nsu033 24622213 PMC4994848

[B45] Wout D. A. Shih M. J. Jackson J. S. Sellers R. M. (2009). Targets as perceivers: how people determine when they will be negatively stereotyped. J. Pers. Soc. Psychol. 96, 349–362. 10.1037/a0012880 19159136 PMC2791406

[B46] Wraga M. Helt M. Jacobs E. Sullivan K. (2007). Neural basis of stereotype-induced shifts in women's mental rotation performance. Soc. Cogn. Affect. Neurosci. 2, 12–19. 10.1093/scan/nsl041 18985116 PMC2555429

[B47] Wu X. Yang W. Tong D. Sun J. Chen Q. Wei D.. (2015). A meta-analysis of neuroimaging studies on divergent thinking using activation likelihood estimation. Hum. Brain Mapp. 36, 2703–2718. 25891081 10.1002/hbm.22801 PMC6869224

[B48] Zhang Q. Shu Y. Li X. Xiong C. Li P. Pang Y.. (2019). Resting-state functional magnetic resonance study of primary open-angle glaucoma based on voxelwise brain network degree centrality. Neurosci. Lett. 712:134500. 10.1016/j.neulet.2019.134500 31557522

[B49] Zuo X. N. Ehmke R. Mennes M. Imperati D. Castellanos F. X. Sporns O.. (2012). Network centrality in the human functional connectome. Cerebral Cortex 22, 1862–1875. 10.1093/cercor/bhr269 21968567

[B50] Zuo X. N. Xing X. X. (2014). Test-retest reliabilities of resting-state FMRI measurements in human brain functional connectomics: a systems neuroscience perspective. Neurosci. Biobehav. Rev. 45, 100–118. 10.1016/j.neubiorev.2014.05.009 24875392

